# Correction to: Physiological plasticity in elephants: highly dynamic glucocorticoids in African and Asian elephants

**DOI:** 10.1093/conphys/coad107

**Published:** 2024-01-03

This is a correction to: Sanjeeta Sharma Pokharel, Janine L Brown, Physiological plasticity in elephants: highly dynamic glucocorticoids in African and Asian elephants, Conservation Physiology, Volume 11, Issue 1, 2023, coad088, https://doi.org/10.1093/conphys/coad088.

The originally published version of this manuscript contained errors in the author affiliations.

These errors have been corrected.

